# ‘*Halomonas saudii*’ sp. nov., a new bacterial species isolated from marine plant *Halocnemum strobilaceum*

**DOI:** 10.1016/j.nmni.2016.11.007

**Published:** 2016-11-15

**Authors:** F. Bibi, M. Yasir, S.A. Alvi, E.I. Azhar, A.A.K. Al-Ghamdi, A.M. Abuzenadah, D. Raoult, E. Angelakis

**Affiliations:** 1) Special Infectious Agents Unit, King Fahd Medical Research Center, Jeddah, Saudi Arabia; 2) Department of Medical Laboratory Technology, Faculty of Applied Medical Sciences, Jeddah, Saudi Arabia; 3) Center of Excellence in Genomic Medicine Research (CEGMR), King Abdulaziz University, Jeddah, Saudi Arabia; 4) KACST Technology Innovation Center in Personalized Medicine, King Abdulaziz University, Jeddah, Saudi Arabia; 5) Unité de Recherche sur les Maladies Infectieuses et Tropicales Emergentes, URMITE CNRS-IRD 198 UMR 6236, Aix-Marseille Université, Faculté de Médecine, Marseille, France

**Keywords:** Culturomics, ‘*Halomonas saudii*’ sp. nov., *Halocnemum strobilaceum*, New species, Saudi Arabia

## Abstract

We report here the main characteristics of ‘*Halomonas saudii*’ strain Saudii DR2 (CSUR P2512), a new species of the *Halomonas* genus that was isolated from a rhizosphere of *Halocnemum strobilaceum* in April 2015.

As a part of a wider culturomics [1] and metagenomics study [2] in Saudi Arabia, we isolated a new bacterium, strain Saudii DR2, from a rhizosphere of *Halocnemum strobilaceum* in April 2015. No identification was obtained for the strain Saudii DR2 using systematic matrix-assisted desorption ionization–time of flight mass spectrometry (MALDI-TOF MS) screening on a MicroFlex spectrometer (Bruker Daltonics, Bremen, Germany). The strain Saudii DR2 was cultured in a homemade liquid medium [3] incubated for 2 days in an aerobic atmosphere at 37°C. Optimal growth for this strain was obtained at 37°C at pH 7; strain Saudii DR2 is halotolerant and tolerates NaCl concentration up to 20%. Saudii DR2 is a Gram-negative bacterium, is motile non–spore forming and does not exhibit catalase or oxidase activities. The growing colonies on our homemade culture medium were orange, circular, entire, smooth and convex, with a diameter of 1.0 to 2.0 mm.

The complete 16S rRNA gene was sequenced using fD1-rP2 primers as previously described and a 3130-XL sequencer (Applied Biosciences, Saint Aubin, France) [4]. The strain Saudii DR2 exhibited a 98.3% sequence similarity with *Halomonas xianhensis* (GenBank accession no. NR116016.1), which was the phylogenetically closest species with standing in nomenclature (Fig. 1). Consequently, it putatively classifies the strain Saudii DR2 as a new member of the genus *Halomonas* within the family *Halomonadaceae* in the phylum *Proteobacteria. H. xianhensis* was first described by Zhao *et al*. [5] in 2012 as an aerobic, Gram-negative, short rod-shaped, moderately halophilic bacterium. So far, the genus *Halomonas* includes more than 80 species with validly published names [6]. Strain Saudii DR2 exhibited a 16S rRNA gene sequence divergence of >1.3% with *H. xianhensis*, the closest related species with standing in nomenclature, which classifies it as a new representative of the *Halomonas* genus isolated from *H. strobilaceum.* As a result, we propose the creation of ‘*Halomonas saudii*’ sp. nov., and the strain Saudii DR2 as the type strain.

## MALDI-TOF MS spectrum accession number

The MALDI-TOF MS spectrum of Saudii DR2 is available online (http://www.mediterranee-infection.com/article.php?laref=256&titre=urms-database).

## Nucleotide sequence accession number

The 16S rRNA gene sequence of the strain Saudii DR2 was deposited in GenBank under accession number LT 558840.1.

## Deposit in a culture collection

Strain Saudii DR2 was deposited in the Collection de Souches de l'Unité des Rickettsies (CSUR, WDCM 875) under number P2512.

## Figures and Tables

**Fig. 1:**
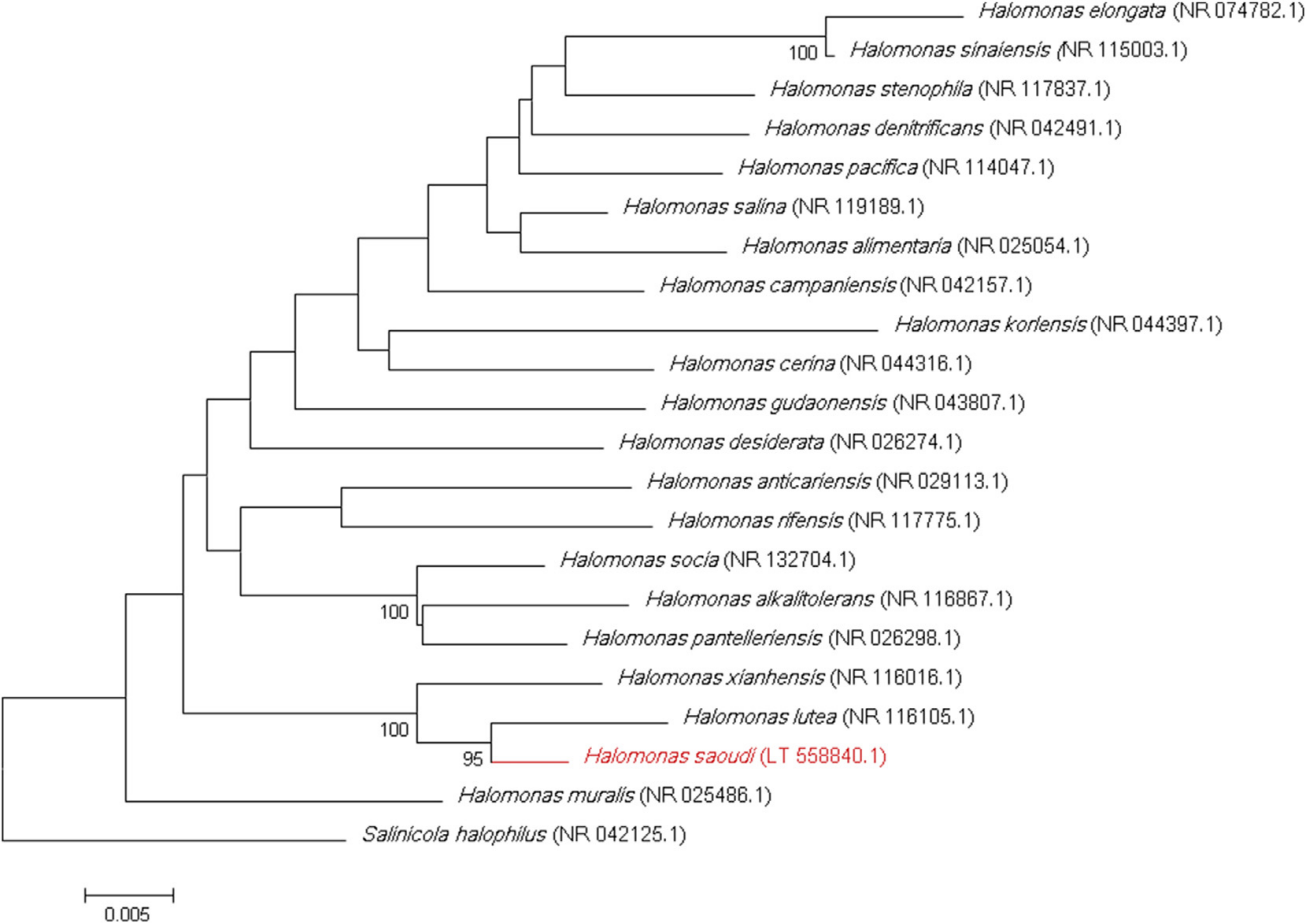
Phylogenetic tree highlighting position of ‘*Halomonas saudii*’ (in red) relative to other phylogenetically closest members of *Halomonas* genus. Number at node is percentage of bootstrap value obtained by repeating analysis 500 times to generate majority consensus tree. Only values >95% were displayed. Scale bar represents 2% nucleotide sequence divergence.
